# Performance of statistical models to predict mental health and substance abuse cost

**DOI:** 10.1186/1471-2288-6-53

**Published:** 2006-10-26

**Authors:** Maria Montez-Rath, Cindy L Christiansen, Susan L Ettner, Susan Loveland, Amy K Rosen

**Affiliations:** 1 Boston University School of Public Health, Department of Biostatistics, Boston, Massachusetts, USA; 2 Center for Health Quality, Outcomes and Economic Research, Bedford VAMC, Bedford, Massachusetts, USA; 3 Boston University School of Public Health, Department of Health Services, Boston, Massachusetts, USA; 4 University of California, Division of General Internal Medicine and Health Services Research, David Geffen School of Medicine, Los Angeles, California, USA

## Abstract

**Background:**

Providers use risk-adjustment systems to help manage healthcare costs. Typically, ordinary least squares (OLS) models on either untransformed or log-transformed cost are used. We examine the predictive ability of several statistical models, demonstrate how model choice depends on the goal for the predictive model, and examine whether building models on samples of the data affects model choice.

**Methods:**

Our sample consisted of 525,620 Veterans Health Administration patients with mental health (MH) or substance abuse (SA) diagnoses who incurred costs during fiscal year 1999. We tested two models on a transformation of cost: a Log Normal model and a Square-root Normal model, and three generalized linear models on untransformed cost, defined by distributional assumption and link function: Normal with identity link (OLS); Gamma with log link; and Gamma with square-root link. Risk-adjusters included age, sex, and 12 MH/SA categories. To determine the best model among the entire dataset, predictive ability was evaluated using root mean square error (RMSE), mean absolute prediction error (MAPE), and predictive ratios of predicted to observed cost (PR) among deciles of predicted cost, by comparing point estimates and 95% bias-corrected bootstrap confidence intervals. To study the effect of analyzing a random sample of the population on model choice, we re-computed these statistics using random samples beginning with 5,000 patients and ending with the entire sample.

**Results:**

The Square-root Normal model had the lowest estimates of the RMSE and MAPE, with bootstrap confidence intervals that were always lower than those for the other models. The Gamma with square-root link was best as measured by the PRs. The choice of best model could vary if smaller samples were used and the Gamma with square-root link model had convergence problems with small samples.

**Conclusion:**

Models with square-root transformation or link fit the data best. This function (whether used as transformation or as a link) seems to help deal with the high comorbidity of this population by introducing a form of interaction. The Gamma distribution helps with the long tail of the distribution. However, the Normal distribution is suitable if the correct transformation of the outcome is used.

## Background

The proportion of patients with Mental Health (MH) and Substance Abuse (SA) disorders in the Department of Veterans Affairs (VA) is higher than in other health care systems [1,2]. In fiscal year 1999 (FY99) (October 1, 1998 – September 30, 1999), approximately 29% of the patients were diagnosed with a MH/SA disorder. Moreover, while the average number of unique diagnoses (including physical and psychiatric) in the general VA population is 9[3], it is about 14 for patients with MH/SA disorders. Because these patients are most likely to have high resource utilization and consumption, adequate risk-adjustment models are needed to accurately predict MH/SA costs and to better allocate resources among competing facilities and networks within the VA.

Cost data typically present challenges because of their skewness and high percentage of zeros. Two-part models have been suggested as a method for solving the problem where data contain a high percentage of zero costs[4,5]. However, obtaining good predictions with the conditional model (the second part of the two-part model) is crucial to the entire modeling process. Generalized linear models (GLMs) are becoming increasingly more popular but, depending on the choice of distribution and link function, estimated effects of risk-adjustment measures may not be additive or multiplicative, and thus, may be difficult to understand and interpret. A commonly used model for cost data is the Log Normal model. Here, the cost is transformed into a logarithmic scale and ordinary least squares (OLS) is performed on this new variable making the effects multiplicative on costs. However, transforming the dependent variable introduces retransformation problems related to the original scale[6-8].

The method most recommended for predicting cost in the health services literature, such as in "Risk Adjustment for Measuring Health Outcomes," [9] is OLS on untransformed cost. Transformation of the outcome or use of GLMs are suggested as ways to handle extreme observations but the authors argue that it is important to keep transparent modeling logic and that OLS is relatively transparent. Although OLS offers transparency with respect to easily understood additive effects, potential prediction problems, e.g., negative cost estimates, are not as transparent to the user of these systems. Also, while OLS yields consistent estimates, it is less efficient than models that explicitly take the skewness into account[7,10].

Sample sizes in many health economic cost studies presented to date vary widely, ranging from a low of 45[11] to a high of 125,109[5]. Austin et al.[12] used a sample of 1,959 patients while Ai and Norton[13] and Buntin and Zaslavsky[14] used samples of approximately 10,000 people. Mullahy[15] used a sample of approximately 30,000 individuals. Our sample of about 500,000 patients is therefore quite large compared to the samples used in the above studies. Building a good predictive risk-adjustment model requires large data sets and we found substantial variation in sample sizes in model-building risk-adjustment studies: from 35,000 to more than a million[16-21]. Kilian et al.[22] suggest that more studies are needed with larger sample sizes and we found no other study that analyzes the implications of having smaller samples.

Two questions of interest arise: 1) Which statistical model works best for our sample of over 500,000 patients if the criteria for the prediction model are low root mean square error (RMSE), low mean absolute prediction error (MAPE), and predictive ratio (PR) around 1 for the entire range of patient costs? and 2) Using these criteria, would the best model for the entire sample be chosen as best if small sample sizes are used? The focus in this paper is on methods for assessing overall model fit and not on the specific effects of the independent variables contained in the risk-adjustment system.

## Methods

### Data description

VA administrative databases were used to select all veterans with a MH/SA diagnosis (ICD-9-CM codes 290.00–312.99, 316) and who used VA healthcare services in FY99. All inpatient and outpatient diagnoses from FY99 were mapped into twelve MH/SA categories (Dementia/Alzheimer's Disease, Alcohol Disorder, Drug Disorder, Schizophrenia, Other Psychoses, Bipolar Disorder, Major Depression, Other Depression, Posttraumatic Stress Disorder (PTSD), Anxiety Disorder, Adjustment Disorder, and Personality Disorder). These categories are used in the VA for mental health performance monitoring[23]. Depending on the diagnoses assigned, a patient can be assigned to more than one MH/SA category.

The majority of VA patients do not pay for their care. The Health Economics Resource Center (HERC), a national VA center, has created estimated costs at the visit/stay level using patient utilization data and cost data at the VA department level. In order to calculate MH/SA costs, we obtained all specialty MH/SA visits or any non-specialty visit (e.g., primary care) for which a MH/SA diagnosis had been assigned. Inpatient drug costs also are included in a patient's annual cost value. A thorough description of the cost data is found in Rosen et al.[24].

### Model specification

Estimates from the models presented in Table 1 and explained in more detail in the next section, were computed using robust weighted generalized linear models. All regression analyses were performed using Stata v8.2, in particular, the glm command with the robust option. This study included individuals with only positive costs; these individuals were part of a development sample used in the study by Rosen et al. [24]. Our development sample comprised 60% of the total population (in Rosen et al. [24]), and included 525,620 individuals having positive cost. The independent variables included 20 age/sex categories and 12 MH/SA categories. Corrections were necessary in order to take into account patients who died during the year and for whom full year data did not exist. Robust regression was performed with the cluster option, where the clusters were medical facilities where patients received care. A patient was assigned to only one facility, defined by the last non-missing facility recorded during FY99. Adjustments were made in all models that forced the predicted mean to equal the observed mean (CN Morris Smearing). Each predicted value was multiplied by a factor equal to the observed sum divided by the predicted sum. Given that the VHA system operates under budgetary constraints set by Congress, it is reasonable to incorporate this property into all models.

**Table 1:** Model's description

Model Name	Dependent Variable	Model Specification			
		
		Family	Link	v(*μ*)	Equation
Normal with identity link (OLS)	*cost*	Normal	Identity	1	*E*(*Y*) = *xβ*
Log Normal*	*ln(cost)*	Normal	Identity	1	*E*{*ln*(*Y*)} = *xβ*
Sqrt Normal*	cost MathType@MTEF@5@5@+=feaafiart1ev1aaatCvAUfKttLearuWrP9MDH5MBPbIqV92AaeXatLxBI9gBaebbnrfifHhDYfgasaacH8akY=wiFfYdH8Gipec8Eeeu0xXdbba9frFj0=OqFfea0dXdd9vqai=hGuQ8kuc9pgc9s8qqaq=dirpe0xb9q8qiLsFr0=vr0=vr0dc8meaabaqaciaacaGaaeqabaqabeGadaaakeaadaGcaaqaaGqaciab=ngaJjab=9gaVjab=nhaZjabdsha0bWcbeaaaaa@325C@	Normal	Identity	1	*E*{Y MathType@MTEF@5@5@+=feaafiart1ev1aaatCvAUfKttLearuWrP9MDH5MBPbIqV92AaeXatLxBI9gBaebbnrfifHhDYfgasaacH8akY=wiFfYdH8Gipec8Eeeu0xXdbba9frFj0=OqFfea0dXdd9vqai=hGuQ8kuc9pgc9s8qqaq=dirpe0xb9q8qiLsFr0=vr0=vr0dc8meaabaqaciaacaGaaeqabaqabeGadaaakeaadaGcaaqaaiabdMfazbWcbeaaaaa@2E02@} = *xβ*
Gamma with log link	*cost*	Gamma	Log	*μ*^2^	*ln*{*E*(*Y*)} = *xβ*
Gamma with square-root link	*cost*	Gamma	Square Root	*μ*^2^	E(Y) MathType@MTEF@5@5@+=feaafiart1ev1aaatCvAUfKttLearuWrP9MDH5MBPbIqV92AaeXatLxBI9gBaebbnrfifHhDYfgasaacH8akY=wiFfYdH8Gipec8Eeeu0xXdbba9frFj0=OqFfea0dXdd9vqai=hGuQ8kuc9pgc9s8qqaq=dirpe0xb9q8qiLsFr0=vr0=vr0dc8meaabaqaciaacaGaaeqabaqabeGadaaakeaadaGcaaqaaiabdweafjabcIcaOiabdMfazjabcMcaPaWcbeaaaaa@30C7@ = *xβ*

* Transformation of dependent variable.

Note: *μ *= E(y) and V(*μ*) is variance function.

### Models

Cost data are non-negative and usually right-skewed. These characteristics guide our choice of models for comparison. In addition, we consider models commonly used in the literature.

Ordinary least squares (OLS) regression on cost is the most common model used. It is the easiest to understand because the independent variables have additive effects:

*E*{**Y**} = **X***β *    (1)

where **Y **are independent Normal variables with constant variance *σ*^2^. Predictions are given in the original scale y^
 MathType@MTEF@5@5@+=feaafiart1ev1aaatCvAUfKttLearuWrP9MDH5MBPbIqV92AaeXatLxBI9gBaebbnrfifHhDYfgasaacH8akY=wiFfYdH8Gipec8Eeeu0xXdbba9frFj0=OqFfea0dXdd9vqai=hGuQ8kuc9pgc9s8qqaq=dirpe0xb9q8qiLsFr0=vr0=vr0dc8meaabaqaciaacaGaaeqabaqabeGadaaakeaacuWG5bqEgaqcaaaa@2E37@ = *x*β^
 MathType@MTEF@5@5@+=feaafiart1ev1aaatCvAUfKttLearuWrP9MDH5MBPbIqV92AaeXatLxBI9gBaebbnrfifHhDYfgasaacH8akY=wiFfYdH8Gipec8Eeeu0xXdbba9frFj0=OqFfea0dXdd9vqai=hGuQ8kuc9pgc9s8qqaq=dirpe0xb9q8qiLsFr0=vr0=vr0dc8meaabaqaciaacaGaaeqabaqabeGadaaakeaaiiGacuWFYoGygaqcaaaa@2E64@. A property of all OLS models is that mean predicted y always equals mean observed y and therefore no other adjustement needs to be made. Because the Normal distribution ranges from minus infinity to plus infinity, negative predictions are possible when applying this model.

Even though OLS is very popular, it does not directly deal with the two characteristics of cost data: non-negative and right-skewed. Transformation of the dependent variable can sometimes solve these issues. In this paper we tested two transformations: log and square root transformation. The first case is commonly called a Log Normal model and the independent variables have additive effects on the log scale:

*E*{**ln**(**Y**)} = **X***β *    (2)

where **ln**(**Y**) are independent Normal variables with constant variance *σ*^2^. Retransformation is necessary in order to get predictions on the original scale. However, direct retransformation y^=exβ^
 MathType@MTEF@5@5@+=feaafiart1ev1aaatCvAUfKttLearuWrP9MDH5MBPbIqV92AaeXatLxBI9gBaebbnrfifHhDYfgasaacH8akY=wiFfYdH8Gipec8Eeeu0xXdbba9frFj0=OqFfea0dXdd9vqai=hGuQ8kuc9pgc9s8qqaq=dirpe0xb9q8qiLsFr0=vr0=vr0dc8meaabaqaciaacaGaaeqabaqabeGadaaakeaacuWG5bqEgaqcaiabg2da9iabdwgaLnaaCaaaleqabaGaemiEaGhcciGaf8NSdiMbaKaaaaaaaa@33EE@ gives biased estimates because this assumes that *E*{**ln**(**Y**)} = **ln**{*E*(**Y**)}, which is not usually true. Several adjustments have been recommended to deal with this problem [6,7]. Forcing the mean predicted to equal mean observed and assuming the error term to be homoscedastic corrects the bias predictions at the population-level. It should also be noted that after retransformation all the additive effects in the log scale become multiplicative effects in the original scale. Final predictions in this model are given by

y^=s⋅exβ^     (3)
 MathType@MTEF@5@5@+=feaafiart1ev1aaatCvAUfKttLearuWrP9MDH5MBPbIqV92AaeXatLxBI9gBaebbnrfifHhDYfgasaacH8akY=wiFfYdH8Gipec8Eeeu0xXdbba9frFj0=OqFfea0dXdd9vqai=hGuQ8kuc9pgc9s8qqaq=dirpe0xb9q8qiLsFr0=vr0=vr0dc8meaabaqaciaacaGaaeqabaqabeGadaaakeaacuWG5bqEgaqcaiabg2da9iabdohaZjabgwSixlabdwgaLnaaCaaaleqabaGaemiEaGhcciGaf8NSdiMbaKaaaaGccaWLjaGaaCzcamaabmaabaGaeG4mamdacaGLOaGaayzkaaaaaa@3B72@

where s=∑y/∑exβ^
 MathType@MTEF@5@5@+=feaafiart1ev1aaatCvAUfKttLearuWrP9MDH5MBPbIqV92AaeXatLxBI9gBaebbnrfifHhDYfgasaacH8akY=wiFfYdH8Gipec8Eeeu0xXdbba9frFj0=OqFfea0dXdd9vqai=hGuQ8kuc9pgc9s8qqaq=dirpe0xb9q8qiLsFr0=vr0=vr0dc8meaabaqaciaacaGaaeqabaqabeGadaaakeaacqWGZbWCcqGH9aqpdaaeabqaaiabdMha5jabc+caVmaaqaeabaGaemyzau2aaWbaaSqabeaacqWG4baEiiGacuWFYoGygaqcaaaaaeqabeqdcqGHris5aaWcbeqab0GaeyyeIuoaaaa@3A50@

Square-root transformation models are similar to the Log Normal model in that retransformation is necessary to bring prediction to the original scale. In this case the model is specified as:

*E*{Y
 MathType@MTEF@5@5@+=feaafiart1ev1aaatCvAUfKttLearuWrP9MDH5MBPbIqV92AaeXatLxBI9gBaebbnrfifHhDYfgasaacH8akY=wiFfYdH8Gipec8Eeeu0xXdbba9frFj0=OqFfea0dXdd9vqai=hGuQ8kuc9pgc9s8qqaq=dirpe0xb9q8qiLsFr0=vr0=vr0dc8meaabaqaciaacaGaaeqabaqabeGadaaakeaadaGcaaqaaGqabiab=LfazbWcbeaaaaa@2E08@} = **X***β *    (4)

where Y
 MathType@MTEF@5@5@+=feaafiart1ev1aaatCvAUfKttLearuWrP9MDH5MBPbIqV92AaeXatLxBI9gBaebbnrfifHhDYfgasaacH8akY=wiFfYdH8Gipec8Eeeu0xXdbba9frFj0=OqFfea0dXdd9vqai=hGuQ8kuc9pgc9s8qqaq=dirpe0xb9q8qiLsFr0=vr0=vr0dc8meaabaqaciaacaGaaeqabaqabeGadaaakeaadaGcaaqaaGqabiab=LfazbWcbeaaaaa@2E08@ are independent Normal variables with constant variance *σ*^2^. Direct retransformation gives y^
 MathType@MTEF@5@5@+=feaafiart1ev1aaatCvAUfKttLearuWrP9MDH5MBPbIqV92AaeXatLxBI9gBaebbnrfifHhDYfgasaacH8akY=wiFfYdH8Gipec8Eeeu0xXdbba9frFj0=OqFfea0dXdd9vqai=hGuQ8kuc9pgc9s8qqaq=dirpe0xb9q8qiLsFr0=vr0=vr0dc8meaabaqaciaacaGaaeqabaqabeGadaaakeaacuWG5bqEgaqcaaaa@2E37@ = (*x*β^
 MathType@MTEF@5@5@+=feaafiart1ev1aaatCvAUfKttLearuWrP9MDH5MBPbIqV92AaeXatLxBI9gBaebbnrfifHhDYfgasaacH8akY=wiFfYdH8Gipec8Eeeu0xXdbba9frFj0=OqFfea0dXdd9vqai=hGuQ8kuc9pgc9s8qqaq=dirpe0xb9q8qiLsFr0=vr0=vr0dc8meaabaqaciaacaGaaeqabaqabeGadaaakeaaiiGacuWFYoGygaqcaaaa@2E64@)^2 ^and after forcing mean predicted to equal mean observed, final predictions are given by

y^=s⋅(xβ^)2     (5)
 MathType@MTEF@5@5@+=feaafiart1ev1aaatCvAUfKttLearuWrP9MDH5MBPbIqV92AaeXatLxBI9gBaebbnrfifHhDYfgasaacH8akY=wiFfYdH8Gipec8Eeeu0xXdbba9frFj0=OqFfea0dXdd9vqai=hGuQ8kuc9pgc9s8qqaq=dirpe0xb9q8qiLsFr0=vr0=vr0dc8meaabaqaciaacaGaaeqabaqabeGadaaakeaacuWG5bqEgaqcaiabg2da9iabdohaZjabgwSixlabcIcaOiabdIha4HGaciqb=j7aIzaajaGaeiykaKYaaWbaaSqabeaacqaIYaGmaaGccaWLjaGaaCzcamaabmaabaGaeGynaudacaGLOaGaayzkaaaaaa@3CC7@

where s=∑y/∑(xβ^)2
 MathType@MTEF@5@5@+=feaafiart1ev1aaatCvAUfKttLearuWrP9MDH5MBPbIqV92AaeXatLxBI9gBaebbnrfifHhDYfgasaacH8akY=wiFfYdH8Gipec8Eeeu0xXdbba9frFj0=OqFfea0dXdd9vqai=hGuQ8kuc9pgc9s8qqaq=dirpe0xb9q8qiLsFr0=vr0=vr0dc8meaabaqaciaacaGaaeqabaqabeGadaaakeaacqWGZbWCcqGH9aqpdaaeabqaaiabdMha5jabc+caVmaaqaeabaGaeiikaGIaemiEaGhcciGaf8NSdiMbaKaacqGGPaqkdaahaaWcbeqaaiabikdaYaaaaeqabeqdcqGHris5aaWcbeqab0GaeyyeIuoaaaa@3BA1@. Squaring *x*β^
 MathType@MTEF@5@5@+=feaafiart1ev1aaatCvAUfKttLearuWrP9MDH5MBPbIqV92AaeXatLxBI9gBaebbnrfifHhDYfgasaacH8akY=wiFfYdH8Gipec8Eeeu0xXdbba9frFj0=OqFfea0dXdd9vqai=hGuQ8kuc9pgc9s8qqaq=dirpe0xb9q8qiLsFr0=vr0=vr0dc8meaabaqaciaacaGaaeqabaqabeGadaaakeaaiiGacuWFYoGygaqcaaaa@2E64@ implicitly introduces two-way interactions into the model.

Generalized linear models (GLMs) are a class of models of the form *g*{*E*(**Y**)} = *xβ*, where g is called the link function and Y has some distribution F[25]. The ordinary least squares model is equivalent to a GLM where the distribution function is Normal and the link function is the identity. These models allow regression of several different statistical distributions like Gamma or Binomial. Typically they are well defined by specifying a mean and a variance function, as illustrated in Table 1, which are the first two moments of any distribution. In contrast to the models where the dependent variable is transformed, retransformation is not a problem and the estimated mean is given directly by applying the inverse of the above function g:

y^=g−1(xβ^)     (6)
 MathType@MTEF@5@5@+=feaafiart1ev1aaatCvAUfKttLearuWrP9MDH5MBPbIqV92AaeXatLxBI9gBaebbnrfifHhDYfgasaacH8akY=wiFfYdH8Gipec8Eeeu0xXdbba9frFj0=OqFfea0dXdd9vqai=hGuQ8kuc9pgc9s8qqaq=dirpe0xb9q8qiLsFr0=vr0=vr0dc8meaabaqaciaacaGaaeqabaqabeGadaaakeaacuWG5bqEgaqcaiabg2da9iabdEgaNnaaCaaaleqabaGaeyOeI0IaeGymaedaaOGaeiikaGIaemiEaGhcciGaf8NSdiMbaKaacqGGPaqkcaWLjaGaaCzcamaabmaabaGaeGOnaydacaGLOaGaayzkaaaaaa@3B52@

Some authors have recommended the use of the Gamma distribution in cost models [7]. This distribution can deal with the long tail typically present in cost data. In this paper, we test the Gamma model with the two link functions also tested as transformations of the dependent variable. Final predictions for the Gamma models are given by

y^=s⋅g−1xβ^     (7)
 MathType@MTEF@5@5@+=feaafiart1ev1aaatCvAUfKttLearuWrP9MDH5MBPbIqV92AaeXatLxBI9gBaebbnrfifHhDYfgasaacH8akY=wiFfYdH8Gipec8Eeeu0xXdbba9frFj0=OqFfea0dXdd9vqai=hGuQ8kuc9pgc9s8qqaq=dirpe0xb9q8qiLsFr0=vr0=vr0dc8meaabaqaciaacaGaaeqabaqabeGadaaakeaacuWG5bqEgaqcaiabg2da9iabdohaZjabgwSixlabdEgaNnaaCaaaleqabaGaeyOeI0IaeGymaedaaOGaemiEaGhcciGaf8NSdiMbaKaacaWLjaGaaCzcamaabmaabaGaeG4naCdacaGLOaGaayzkaaaaaa@3D5B@

where s=∑y/∑g−1xβ^
 MathType@MTEF@5@5@+=feaafiart1ev1aaatCvAUfKttLearuWrP9MDH5MBPbIqV92AaeXatLxBI9gBaebbnrfifHhDYfgasaacH8akY=wiFfYdH8Gipec8Eeeu0xXdbba9frFj0=OqFfea0dXdd9vqai=hGuQ8kuc9pgc9s8qqaq=dirpe0xb9q8qiLsFr0=vr0=vr0dc8meaabaqaciaacaGaaeqabaqabeGadaaakeaacqWGZbWCcqGH9aqpdaaeabqaaiabdMha5jabc+caVmaaqaeabaGaem4zaC2aaWbaaSqabeaacqGHsislcqaIXaqmaaGccqWG4baEiiGacuWFYoGygaqcaaWcbeqab0GaeyyeIuoaaSqabeqaniabggHiLdaaaa@3C46@ and g is either the log or the square-root functions.

### Model selection and validation

The models' predictive ability was evaluated using the root mean square error (RMSE) and the mean absolute prediction error (MAPE). These are common statistics used to assess models in the risk-adjustment and health economics literature[8,12,14,21]. Defining *y*_*i *_as the observed cost and y^i
 MathType@MTEF@5@5@+=feaafiart1ev1aaatCvAUfKttLearuWrP9MDH5MBPbIqV92AaeXatLxBI9gBaebbnrfifHhDYfgasaacH8akY=wiFfYdH8Gipec8Eeeu0xXdbba9frFj0=OqFfea0dXdd9vqai=hGuQ8kuc9pgc9s8qqaq=dirpe0xb9q8qiLsFr0=vr0=vr0dc8meaabaqaciaacaGaaeqabaqabeGadaaakeaacuWG5bqEgaqcamaaBaaaleaacqWGPbqAaeqaaaaa@2FBE@ as the predicted cost for an individual then

RMSE=1n∑i=1n(yi−y^i)2     (8)
 MathType@MTEF@5@5@+=feaafiart1ev1aaatCvAUfKttLearuWrP9MDH5MBPbIqV92AaeXatLxBI9gBaebbnrfifHhDYfgasaacH8akY=wiFfYdH8Gipec8Eeeu0xXdbba9frFj0=OqFfea0dXdd9vqai=hGuQ8kuc9pgc9s8qqaq=dirpe0xb9q8qiLsFr0=vr0=vr0dc8meaabaqaciaacaGaaeqabaqabeGadaaakeaacqWGsbGucqWGnbqtcqWGtbWucqWGfbqrcqGH9aqpdaGcaaqaamaalaaabaGaeGymaedabaGaemOBa4gaamaaqahabaGaeiikaGIaemyEaK3aaSbaaSqaaiabdMgaPbqabaGccqGHsislcuWG5bqEgaqcamaaBaaaleaacqWGPbqAaeqaaOGaeiykaKYaaWbaaSqabeaacqaIYaGmaaaabaGaemyAaKMaeyypa0JaeGymaedabaGaemOBa4ganiabggHiLdaaleqaaOGaaCzcaiaaxMaadaqadaqaaiabiIda4aGaayjkaiaawMcaaaaa@496C@

and

MAPE=1n∑i=1n|yi−y^i|     (9)
 MathType@MTEF@5@5@+=feaafiart1ev1aaatCvAUfKttLearuWrP9MDH5MBPbIqV92AaeXatLxBI9gBaebbnrfifHhDYfgasaacH8akY=wiFfYdH8Gipec8Eeeu0xXdbba9frFj0=OqFfea0dXdd9vqai=hGuQ8kuc9pgc9s8qqaq=dirpe0xb9q8qiLsFr0=vr0=vr0dc8meaabaqaciaacaGaaeqabaqabeGadaaakeaacqWGnbqtcqWGbbqqcqWGqbaucqWGfbqrcqGH9aqpdaWcaaqaaiabigdaXaqaaiabd6gaUbaadaaeWbqaamaaemaabaGaemyEaK3aaSbaaSqaaiabdMgaPbqabaGccqGHsislcuWG5bqEgaqcamaaBaaaleaacqWGPbqAaeqaaaGccaGLhWUaayjcSdaaleaacqWGPbqAcqGH9aqpcqaIXaqmaeaacqWGUbGBa0GaeyyeIuoakiaaxMaacaWLjaWaaeWaaeaacqaI5aqoaiaawIcacaGLPaaaaaa@4987@

Large values indicate a poor fit. To further test each model's performance, we also looked at predictive ratios (PRs), which are a group-level type of measure [26]. These were computed for deciles of predicted cost. For a decile j, the PR is the ratio of predicted cost to observed cost among patients in each decile. The closer the values are to 1.0, the better the model prediction is within that decile. Following the method by Kilian et al.[22] we computed bias-corrected bootstrap confidence intervals for each one of these measures to help with the comparison of the different models. This was done using the bootstrap command in Stata using 1000 replications and the strata option to allow for independent sampling within each facility.

### Sample size study

Data access problems, costs, and other obstacles often limit the amount of data available for building a risk adjustment model. To study the effect of developing the model on a subset of the data, we examined the effect of sample size on model choice using randomly selected samples of various sizes. We sought to answer the question: is model choice affected by the size of the sample used? We varied sample sizes between 5,000 and the entire sample. However, the results for samples above 80,000 were very stable. For each size, we randomly selected 100 samples, ran all five models, and computed all the statistical measures mentioned above (RMSE, MAPE, and PRs by decile of predicted cost). We compare 95% percentile confidence intervals of each measure within each sample size for all five models.

## Results

The total sample consisted of 525,620 patients; 95% were males, with a mean age of 57 years (SD = 14.0); about 30% were age 65 and older. The dependent variable, total MH/SA cost, had a mean of 2,602 (SD = 11,052), a median equal to 385, and a skewness of 14. The residual plot from the Normal Identity model indicated heteroscedasticity and the QQ plot showed non-normality of the error term. The QQ plot of the residuals from the Log Normal model also indicated non-normality. Predicted values for costs in the Normal Identity model took on negative values for 19.6% of the total sample.

Table 2 summarizes the overall performance of the models including bootstrap confidence intervals (BCIs). The Square-root Normal model had a RMSE of 9,860 and a MAPE value of 2,554, the lowest of all five models. Moreover, its bootstrap confidence intervals were lower than any other BCI. The OLS model and the Gamma with square-root link model had the second best RMSEs, with both estimates and BCIs very close in value. The Gamma with square-root link and the Log Normal model presented the second best estimates for the MAPE, also with very close BCIs. For both measures, the Gamma Log model performed the worst, with a RMSE twice the value of the RMSE for Square-root Normal model and with confidence intervals that are outside the bounds of all other models' BCIs.

**Table 2:** Root mean square error (RMSE) and mean absolute prediction error (MAPE) results obtained for the 5 models run in the full sample of 525,620 patients

Model	RMSE			MAPE		
		
	Estimate	95% Conf.	Int.*	Estimate	95% Conf.	Int.*
Normal with Identity link (OLS)	10,397	10,130	10,657	2,997	2,941	3,052
Log Normal	13,974	13,585	14,352	2,801	2,759	2,840
Sqrt Normal	9,860	9,644	10,070	2,554	2,514	2,592
Gamma with log link	21,374	20,246	22,552	3,324	3,249	3,395
Gamma with square-root link	10,434	10,193	10,708	2,797	2,744	2,859

* Bias-corrected bootstrap confidence interval

**Figure 1:**
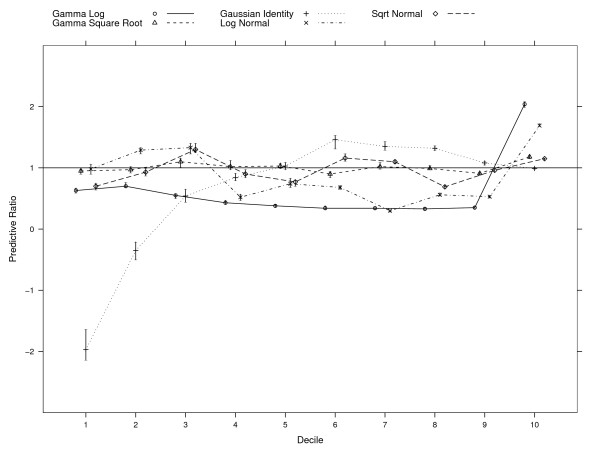
**Predictive ratios (PR) per decile of predicted cost in full sample (N = 525,620)**. PR is computed as the ratio of predicted cost to observed cost for deciles of predicted cost. For each decile, PR = 1 when mean predicted cost equals mean observed cost. Also shown, are 95% bias-corrected bootstrap confidence intervals.

Inspection of the PRs among deciles of predicted total MH/SA cost (see Figure 1) indicates that only the Gamma Square Root model performed reasonably well, with PRs close to 1.0 (values ranging from 0.90 to 1.18) across all 10 deciles. Moreover, the BCIs included 1.0 for five of the deciles (deciles 2, 4, 5, 8, and 9). The Normal Identity model (OLS) had two BCIs that included 1.0 (deciles 5 and 10). However, it had negative PRs for the first two deciles because of the 20% negative predictions, and for the remaining eight the values ranged from 0.54 to 1.46. The Log Normal model did well in the first decile, but either underpredicted or overpredicted by more than 25% for the remaining nine deciles (values ranging from 0.30 to 1.69). The Square-root Normal model had values ranging from 0.9 to 1.18, with BCIs that never included 1.0. The Gamma Log model underpredicted substantially in the first nine deciles (values ranging from 0.33 to 0.66) and overpredicted in the top decile (2.04) with BCIs that also never included 1.0.

### Sample size study

The Gamma with square-root link model had convergence problems with the smallest samples. The frequencies of samples for which models did not converge, ranged from 1 (sample with 55,000 patients) to 50 (samples with 5,000 patients) out of 100 replications within each sample size tested. There were three categories for which this problem occurred the most and they were for females in three age groups: 70–74, 80–84, and 85 or older. Each one of these groups had, in the overall sample, 74, 70, and 35 patients, respectively. When resampling, these small groups become extremely small, with categories having only 1 or 2 patients after sampling. Even though the procedure had problems converging when fitting the Gamma with square root link, this did not present a problem for the other four statistical models.

**Figure 2:**
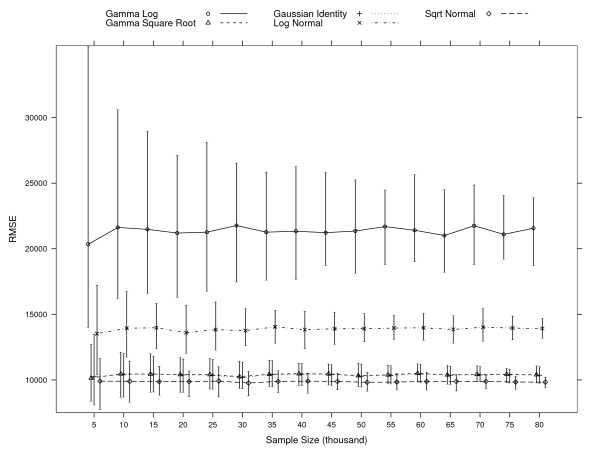
**95% root mean square error (RMSE) percentile intervals per model at each simulation of various sample sizes**. RMSE=1n∑i=1n(yi−y^i)2
 MathType@MTEF@5@5@+=feaafiart1ev1aaatCvAUfKttLearuWrP9MDH5MBPbIqV92AaeXatLxBI9gBaebbnrfifHhDYfgasaacH8akY=wiFfYdH8Gipec8Eeeu0xXdbba9frFj0=OqFfea0dXdd9vqai=hGuQ8kuc9pgc9s8qqaq=dirpe0xb9q8qiLsFr0=vr0=vr0dc8meaabaqaciaacaGaaeqabaqabeGadaaakeaacqWGsbGucqWGnbqtcqWGtbWucqWGfbqrcqGH9aqpdaGcaaqaamaalaaabaGaeGymaedabaGaemOBa4gaamaaqadabaGaeiikaGIaemyEaK3aaSbaaSqaaiabdMgaPbqabaGccqGHsislcuWG5bqEgaqcamaaBaaaleaacqWGPbqAaeqaaOGaeiykaKYaaWbaaSqabeaacqaIYaGmaaaabaGaemyAaKMaeyypa0JaeGymaedabaGaemOBa4ganiabggHiLdaaleqaaaaa@4557@ Large values indicate a poor fit. The simulations at each sample size are based on 100 samples with the exception of the simulations for the Gamma Square Root model. Samples for which the model did not converge are dropped: 50 when sampling 5,000 subjects, 15 for 10,000, 16 for 15,000, 8 for 20,000, 7 for 25,000 and 30,000, 5 for 35,000, and 1 for 50,000 and 55,000.

**Figure 3:**
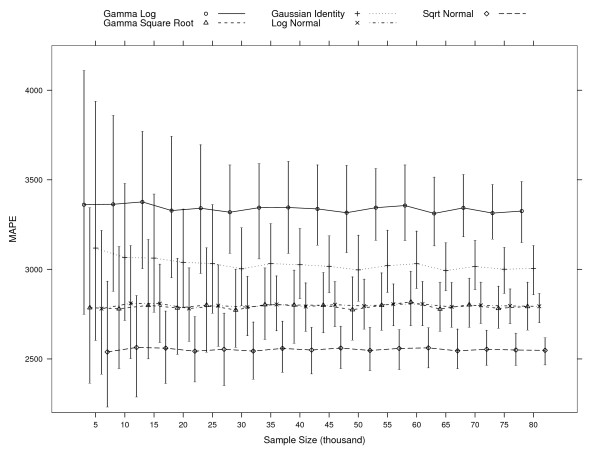
**95% mean absolute prediction error (MAPE) percentile intervals per model at each simulation of various sample sizes**. MAPE=1n∑i=1n|yi−y^i|
 MathType@MTEF@5@5@+=feaafiart1ev1aaatCvAUfKttLearuWrP9MDH5MBPbIqV92AaeXatLxBI9gBaebbnrfifHhDYfgasaacH8akY=wiFfYdH8Gipec8Eeeu0xXdbba9frFj0=OqFfea0dXdd9vqai=hGuQ8kuc9pgc9s8qqaq=dirpe0xb9q8qiLsFr0=vr0=vr0dc8meaabaqaciaacaGaaeqabaqabeGadaaakeaacqWGnbqtcqWGbbqqcqWGqbaucqWGfbqrcqGH9aqpdaWcaaqaaiabigdaXaqaaiabd6gaUbaadaaeWaqaamaaemaabaGaemyEaK3aaSbaaSqaaiabdMgaPbqabaGccqGHsislcuWG5bqEgaqcamaaBaaaleaacqWGPbqAaeqaaaGccaGLhWUaayjcSdaaleaacqWGPbqAcqGH9aqpcqaIXaqmaeaacqWGUbGBa0GaeyyeIuoaaaa@4570@ Large values indicate a poor fit. The simulations at each sample size are based on 100 samples with the exception of the simulations for the Gamma Square Root model. Samples for which the model did not converge are dropped: 50 when sampling 5,000 subjects, 15 for 10,000, 16 for 15,000, 8 for 20,000, 7 for 25,000 and 30,000, 5 for 35,000, and 1 for 50,000 and 55,000.

Figure 2, 3, and 4 shows 95% percentile intervals from each simulation of sample sizes starting at 5,000 up to 80,000 patients, for three measures: RMSE, MAPE, and the predictive ratio for decile 10, which includes 10% of the patients having the highest predicted cost. In Figure 2, the Gamma with square-root link and OLS models have mean estimates and percentile intervals for the RMSE that are very similar for all sample sizes tested. The interval overlap of these models and the one for the Square-root Normal model decreases as the sample size increases. These three models show the lowest RMSE values. The percentile interval for the Log Normal model overlaps with other three intervals for samples with 5,000 patients. The intervals for the Gamma with log link model are always higher than the intervals for the Gamma with square-root link, OLS, and Square-root Normal models. If the criterion for model selection is to choose the one with smallest RMSE, OLS and Gamma with square-root link could also be chosen, besides the Square-root Normal model which gives the smallest RMSE in the full sample.

**Figure 4:**
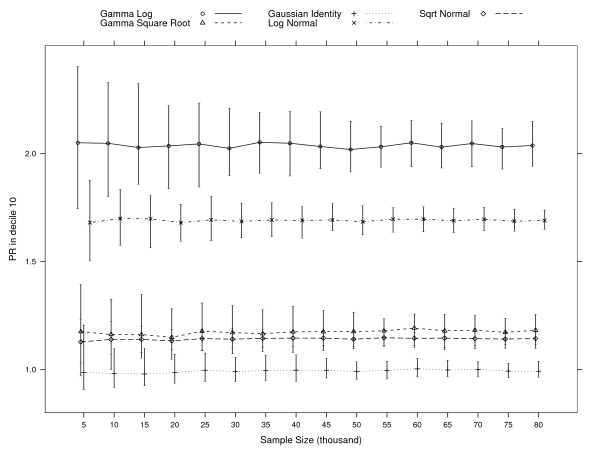
**95% predicted ratio for decile 10 (PR10) percentile intervals per model at each simulation of various sample sizes**. PR is computed as the ratio of predicted cost to observed cost whithin decile 10 of predicted cost. PR = 1 when mean predicted cost equals mean observed cost. The simulations at each sample size are based on 100 samples with the exception of the simulations for the Gamma Square Root model. Samples for which the model did not converge are dropped: 50 when sampling 5000 subjects, 15 for 10,000, 16 for 15,000, 8 for 20,000, 7 for 25,000 and 30,000, 5 for 35,000, and 1 for 50,000 and 55,000.

In Figure 3, we see a bigger overlap between all models. For samples with 5,000 patients, all percentile intervals overlap. The square-root normal model intervals have consistently the smallest lower bound and for samples with at least 15,000 patients the upper bound is always smaller than the average MAPE of all other 4 models. As the sample size increases, the upper bounds of the intervals for the Square-root Normal model become smaller and isolated from the other models. However, for smaller sample sizes, one could pick both the Log Normal and Gamma with square-root link models as giving the lowest MAPE, even though it is the Square-root Normal model that has the smallest MAPE in the full sample.

For samples with 5,000 patients, both the Gamma with square-root link and OLS models have percentile intervals for the predicted ratio in decile 10 that include 1.0 (see Figure 4). This means that for some samples, these models give a predicted mean close to the observed mean for those predicted to be in the top decile. The Square-root Normal model has intervals that overlap more for 5,000 patients and less up to 15,000 patients. However, these intervals are the smallest for all models. Intervals for the Gamma with log link and Log Normal models are always higher than the intervals for all other three models.

## Discussion

This analysis used five statistical models to predict cost for a population of patients with MH/SA disorders in the VA. Several methods for overall model fit, as well as fit within deciles of predicted costs, were used to test the predictive ability of the models. Moreover, a test of sensitivity of model choice to sample size was performed using simulation methods.

Ordinary least squares is often used to regress cost on patient characteristics. The population tested in this study has multiple comorbidities, with some patients (or a large proportion) incurring very high costs. This causes the tail of the distribution of costs to be very right-skewed and residuals from the model are not distributed normally. Nevertheless, even for distributions that account for long tails, often there are not enough observations with extremely high values to estimate the tail accurately.

The sample used in this study is large (more than 10 times larger than what is reported for other studies) and allows for extensive study of how well each of the models predict and also how well they predict for smaller sample sizes. This is of extreme importance, given that in many studies, researchers do not have access to such large datasets or for other reasons cannot analyze data from an entire population.

The Gamma Log model was found to be the worst model in every statistic analyzed. It did particularly poorly for the RMSE, with a value that was more than double the smallest RMSE value corresponding to the Normal Identity model. It also performed poorly for deciles of predicted cost, underpredicting consistently for the first 9 deciles and overpredicting in the 10th decile.

Nixon and Thompson [11] conclude that skewed parametric distributions fit cost data better than the Normal distribution. In our sample, the Normal Identity model performed well, as shown by the measures of the predictions in the full sample. The Normal Identity had the second smallest RMSE and a predictive ratio for the top decile not statistically different from 1. The major problem with this model is the extremely large percentage of negative predictions it generates. If all one is interested in is the overall mean prediction, then the model is adequate. However, if one is interested in individual predictions, such as those patients with small costs, the model is not as good. Nonetheless it performed reasonably well at the top deciles, which is often an important target group for policy makers and disease management planners.

The models tailored to deal with the skewed sample perform reasonably well. In the overall sample, models with square-root transformation or link perform the best. This could be due to the fact that the square root transformation forces a form of interaction among the independent variables that might be needed in this sample because many of the patients have multiple MH/SA conditions. Interactions usually are not used in risk-adjustment systems except for systems that use hierarchies within conditions. However, hierarchies are a limited form of interactions and are designed primarily to avoid double counting specific diagnoses within a disease category, e.g., for a patient with paranoid schizophrenia and psychoses NOS ("not otherwise specified"), only the paranoid schizophrenia is counted. The Square-root Normal model has the smallest MAPE and RMSE that are statistically different from the other models values. The Gamma with square-root link has PRs that are (for each decile) consistently very close to 1.

The Log Normal is a multiplicative model. It does well when assessed on the log scale (not shown here) but after retransformation and even with adjustments, it does poorly. One reason is the fact that we are using a sample of all MH/SA patients, which are, in general, a highly comorbid population within the VA. Those individuals most comorbid are the ones found in the upper deciles. When bringing predictions back to the original scale, the multiplicative effect in this model causes large predictions as evidenced by an extremely high PR in the 10th decile. The overprediction in the 10th decile, together with the fact that we are forcing the mean predicted to equal the mean observed, translates into very poor predictions in the middle deciles.

Simulation results show that even though on average results do not differ from those in the larger sample, gamma models have some convergence problems for smaller sample sizes. However, this problem is directly related to the extremely small number of subjects in certain cells. This can be dealt with by inspecting the data before running the model. In the case of obtaining a sample with very small numbers for certain categories, the investigator should consider combining categories with small cell sizes before deciding that gamma models cannot be run. In the sample presented, the Gamma with square-root link model gives a very good fit in the overall sample; even for the small samples where the model converges, this is a reasonable choice based on the statistics we assessed.

Choosing a parsimonious model is an important statistical practice. This argument often is used to justify the choice of OLS risk-adjustment models. However, parsimony requires that the model be the simplest one possible that also fits the data well. The large percentage of negative predictions from our OLS models invalidates, in this study, this characterization for the OLS model.

More advanced models have been introduced in the literature that are an extension of the GLM models. Basu and Rathouz 2005 [27] introduce a method that directly estimates the link function in a GLM from the data. Manning, Basu, and Mullahy 2005 [28] describe the generalized Gamma models, which include the OLS with Normal error, OLS for Log Normal, and Gamma with a log link as special cases. One limitation of our study is that we compared models using one risk-adjustment system. The population of interest and the goals for the risk-adjustment system dictate our choice of the best model for this setting. So, although the model choice may not generalize to other risk-adjustment systems, the process of defining goals and testing whether the model meets the goals is generalizable to good statistical practice.

## Conclusion

This work provides further statistical information on model performance and model choice for risk-adjustment models used for predicting costs in patients with MH/SA diagnoses. We use one MH/SA risk-adjustment system and compare five different statistical models. We found that the models with square-root transformation or link performed best in the full sample. This function (whether used as transformation or as a link) seems to help deal with the high comorbidity of this population by introducing a form of interaction. The Gamma distribution is modeling the variance better, as seen in better predictions throughout all 10 deciles. However, the Normal distribution is suitable if the correct transformation (square-root in our case) of the outcome is used and this should be true when this method is applied to highly comorbid populations. For smaller samples, the Gamma with square-root link model had problems converging. However, this was directly tied to very small numbers in certain categories and this can be solved by collapsing some of the categories. OLS on untransformed cost and the Log Normal and Square-root Normal model are relatively unaffected by the sample size for the criteria we used, while the GLMs assuming a Gamma distribution are less consistent for smaller sample sizes.

## Authors' contributions

MMR carried out the statistical analysis and drafted the manuscript including the preparation of tables and figures. CLC carried out the modelling and drafted most of the discussion. SL constructed the cost data. SLE and AKR critically revised different sections of the manuscript. All authors contributed to commenting on drafts of the manuscript and have read and approved the final manuscript.

## Pre-publication history

The pre-publication history for this paper can be accessed here:


